# The relative age effect is widespread among European adult professional soccer players but does not affect their market value

**DOI:** 10.1371/journal.pone.0283390

**Published:** 2023-03-23

**Authors:** Eduard Bezuglov, Ryland Morgans, Mikhail Butovskiy, Anton Emanov, Larisa Shagiakhmetova, Bekzhan Pirmakhanov, Zbigniew Waśkiewicz, Artemii Lazarev

**Affiliations:** 1 Sechenov First Moscow State Medical University (Sechenov University), Moscow, Russian Federation; 2 High Performance Sports Laboratory, Moscow Witte University, Moscow, Russian Federation; 3 Academy of Talents, Moscow, Russian Federation; 4 FC Rubin, Kazan, Russian Federation; 5 Smart Recovery Sports Medicine Clinic, Moscow, Russian Federation; 6 Faculty of Medicine and Health Care, Department of Epidemiology, Biostatistics and Evidence-Based Medicine, Al-Farabi Kazakh National University, Almaty, Kazakhstan; 7 FC Kairat, Almaty, Kazakhstan; 8 Jerzy Kukuczka Academy of Physical Education in Katowice, Katowice, Poland; 9 Mount Sinai Hospital, Chicago, IL, United States of America; Instituto Politécnico de Santarém: Instituto Politecnico de Santarem, PORTUGAL

## Abstract

**Background:**

The relative age effect (RAE) is most prevalent in highly competitive youth soccer and persists to a lesser extent in senior soccer. However, it is known that soccer players born in the second half of the year are as successful at senior level, indicating that they are equally talented although under-represented at youth level due to bias during the selection process, in which the emphasis is on more pronounced physical qualities in a specific period of time. Examining the prevalence of the RAE among professional soccer players depending on the level of competition and playing position, as well as analyzing the relationship between the player’s birth quarter and market value, are of scientific interest.

**Methods:**

The dates of birth, playing position, and market value of all adult male soccer players included in the final rosters of teams from the top-division of 54 European countries, listed on www.transfermarkt.com on August 15th, 2020, were analyzed (18,429 soccer players in total). All players were categorized into four groups according to the quarter of birth (Q) and playing position. All teams were further sub-divided in groups depending on the soccer clubs’ level of representation in the UEFA Champions League.

**Results:**

Of 18,429 players, 30.9% were born in Q1, 25.7% in Q2, 23.8% in Q3 and 19.6% in Q4. The number of soccer players born in Q1 was lower in less competitive leagues. The number of players born in Q1 decreased as the level of competition decreased; the highest percentage of these players was observed in clubs that are among the top 50 ranked in UEFA or compete in the most prestigious European championships. The RAE was less pronounced in teams that participate in lower competitive championships. There was no significant difference in market value between players playing position and level of competition when born in different quarters. Although, the most expensive soccer players in the European championships were late-born forwards. Players of various groups differed in their market value.

**Conclusions:**

The RAE is currently prevalent in all the most competitive senior soccer leagues and teams in Europe regardless of playing position. There are no significant differences in market value between players of the same playing position and level of competition when born in different quarters. The most expensive soccer players in the European championships are forwards born in Q4. These findings may indicate that the under-representation of “late-born” soccer players in youth, and then consequently in adult soccer, is not associated with lower talent, but with other factors, possibly based on physiological characteristics and socio-cultural factors. Further measures are needed to mitigate the discriminatory effects of selection bias based on the RAE.

## Introduction

The relative age effect (RAE) refers to the over-representation of athletes born before a specific date for age grouping in various sports comparing to those born after this date [1]. Athletes born relatively late in the selection year potentially suffer a disadvantage during the selection process [2]. In sports where the selection cut-off date is January 1st, the number of athletes born between January and March may be several times higher than the number of athletes born between October and December [3]. Thus the RAE is widespread among young male athletes (age 15–18 years) performing competitively in soccer, athletics, and basketball [1,3–6]. Many studies have examined the wide spread of the RAE among soccer players of different ages and levels of competition [7–10]. Williams et al. showed that in a cohort of FIFA U17 World Cup players approximately 40% were born in the first quarter of the year while only 16% were born in the last three months of the year [11]. Rada et al. further demonstrated that the number of players born in the first month of the calendar year is twice as many of those born in the last month of the year, while the RAE is also widely prevalent in second-tier players [12]. More recent studies also showed that the RAE is evident in elite German [13] and Scottish soccer players [14]. Although, the prevalence of the RAE in older soccer players is not as high when compared to younger players [15].

The main negative factor of the RAE is the "discrimination" of late-born athletes likely related to them being less physically developed (as they are younger) and therefore considered less mature. For example, a study by Romann et al. showed that the difference in 60-meter sprint performance between “early-” and “late-born” athletes aged 8 to 15 years born in one year ranged from 5–10% [16]. However, maturity and the RAE are two independent phenomena, and among early-born and late-born athletes, the ratio of early-, on-time and late-maturing is the same [17], i.e., both early- and late-born athletes can be early- or late-maturing.

Recently, there has also been growing interest in analyzing the relationship between a soccer player’s date of birth and the corresponding market value, although the available data are conflicting and limited. Furley et al. found that in the top 100 soccer players, there were more “early-born” players and these players had a higher average market value [18]. Contrastingly, Doyle et al. analyzed the top 1000 professional UEFA U19 Youth League players and found that the market value of “early-born” players does not exceed the market value of “late-born” players [19]. Thus, it is practically interesting to examine the RAE and its association with the market value in soccer players. This study aimed to analyze the relationship between the player’s birth quarter and their market value and to assess the prevalence of the RAE depending on the competitive level and playing position of European soccer players.

## Materials and methods

The materials used in this article are based on squad data from the top divisions of 54 European countries downloaded from www.transfermarkt.com. Data were publicly available as of September 10^th^, 2020. **The website Transfermarkt (www.transfermarkt.com) has been recently used as a data source for elite soccer studies [2,19–22]. Transfermarkt has information on male soccer players only; female players are not included. This source has previously proven to provide reliable match performance indicators and has been described as a good predictor of real market values [23]. This database has also been used for studies on the RAE. For example, Doyle et al. used the top 1000 soccer player Transfermarkt value in the 2013–2014 season to report that early-born players were worth more [19].**

For data mining, software was used to exploit the PHP Simple HTML DOM Parser library. All publicly accessible pages have been analyzed page by page to obtain information on the player’s date of birth, position on the field, and market value. The collected data was organized into a MySQL database, which was then used to design queries to select and interpret the required data. Local ethics committee approval was not required since publicly available data was utilized. Overall, the analysis included data on 18,429 soccer players from 731 top-division teams playing in 54 European First Leagues from respective countries (there is no First League in the Principality of Liechtenstein). These 54 countries have been divided into four groups based on the level of representation of the country’s soccer clubs in the 2018/2019 UEFA Champions League [24], as this appears to be the most objective method of classifying European club soccer and this classification was made by European soccer authorities (UEFA). **Group 1** included six countries that had the maximum representation of seven clubs in the Champions League, respectively (Spain, England, Germany, Italy, France, and Russia). **Group 2** included nine countries with five teams in the Champions League (Portugal, Belgium, Ukraine, Turkey, Netherlands, Austria, Czech Republic, Greece, and Switzerland). **Group 3** included 35 countries, four teams in Champions League (Denmark, Croatia, Cyprus, Serbia, Scotland, Belarus, Sweden, Norway, Kazakhstan, Poland, Azerbaijan, Israel, Bulgaria, Romania, Slovakia, Slovenia, Hungary, Albania, North Macedonia, Bosnia and Herzegovina, Moldova, Ireland, Finland, Georgia, Malta, Iceland, Wales, Northern Ireland, Montenegro, Estonia, Faroe Islands, Luxembourg, Armenia, Latvia, and Lithuania). **Group 4** includes four countries with one to three teams in the Champions League (San Marino, Andorra, Kosovo, Gibraltar). The top 50 European teams according to the UEFA ranking 2019/2020 [25] were analyzed separately as a group of "best teams" (BT) and was the group of the highest competitive level.

All players were divided into four groups according to their month of birth:

- Players born in the first quarter of the year (January, February, March) (Q1, early-born)
- Players born in the second quarter of the year (April, May, June) (Q2)
- Players born in the third quarter
- of the year (July, August, September) (Q3)
- Players born in the fourth quarter of the year (October, November, December) (Q4, late-born).

The RAE was defined as a higher relative sample size in the first quarter compared to other quarters by date of birth.

Players were also grouped by position, Goal-keepers (n = 2075), defenders (n = 5884), midfielders (n = 7826), and forwards (n = 2644).

### Statistical analysis

Data were stored in MS Excel. Analysis was performed with SPSS Statistics v.23.0 software (IBM). The chi-square test was used to compare the number of “early-born” and “late-born” players in different groups. The unconditional maximum likelihood estimation and normal approximation (Wald) CI method were used to calculate odds ratios and 95% confidence intervals. The Kruskal-Wallis test was used to compare market value for players born in Q1-4 and between groups. For statistically significant differences we also performed post-hoc pairwise comparisons using the Dunn test with Holm adjustment. Significance level was kept at 5%.

## Results

**Fig 1:**
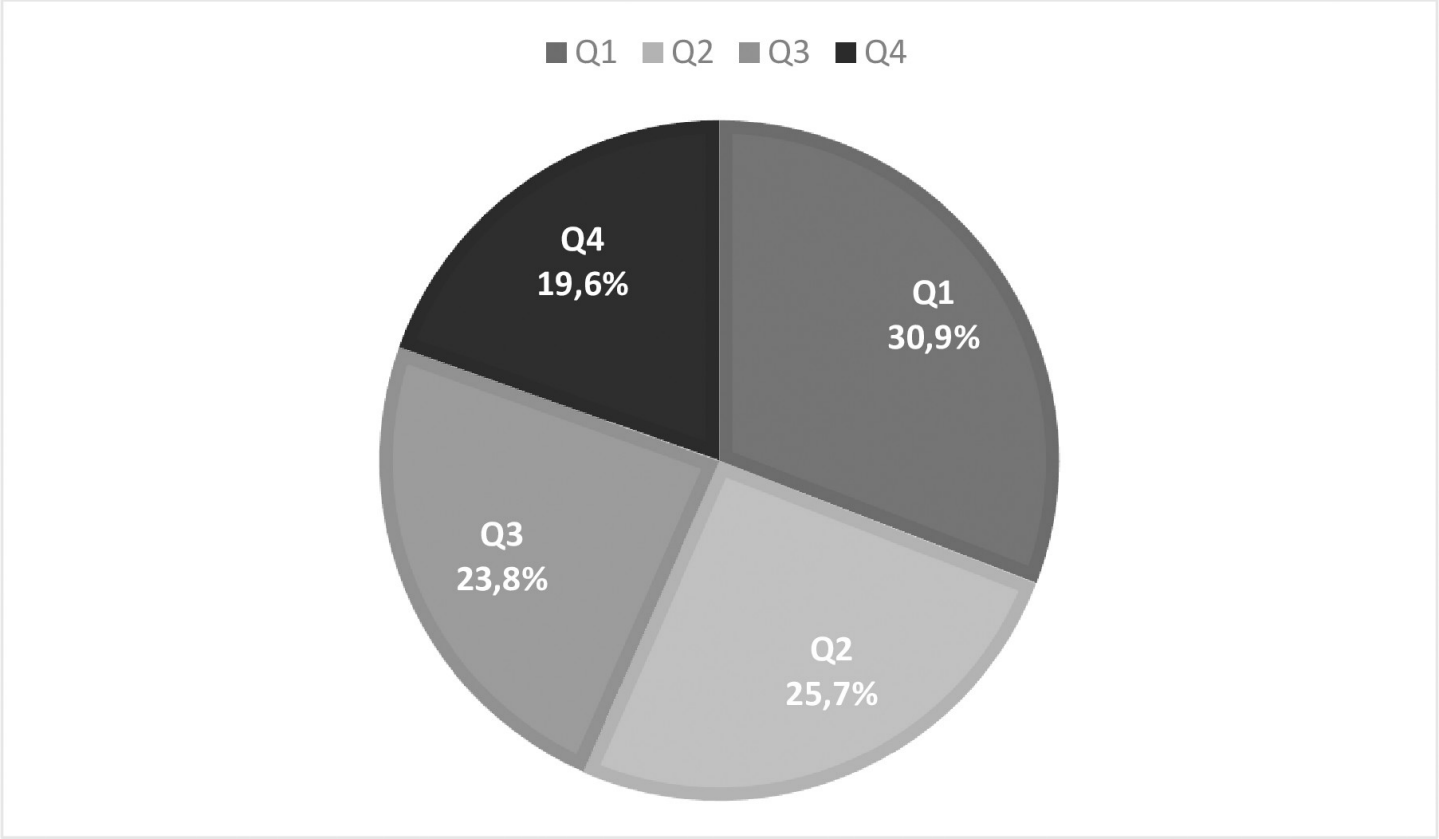
Distribution of players in all European championships by the quarter of birth.

18,429 players competed in 731 top division teams in 54 European countries. Of these, 30.9% (5,688) were born in Q1 **and** 19.6% (3,609) in Q4 (Fig 1).

**Fig 2:**
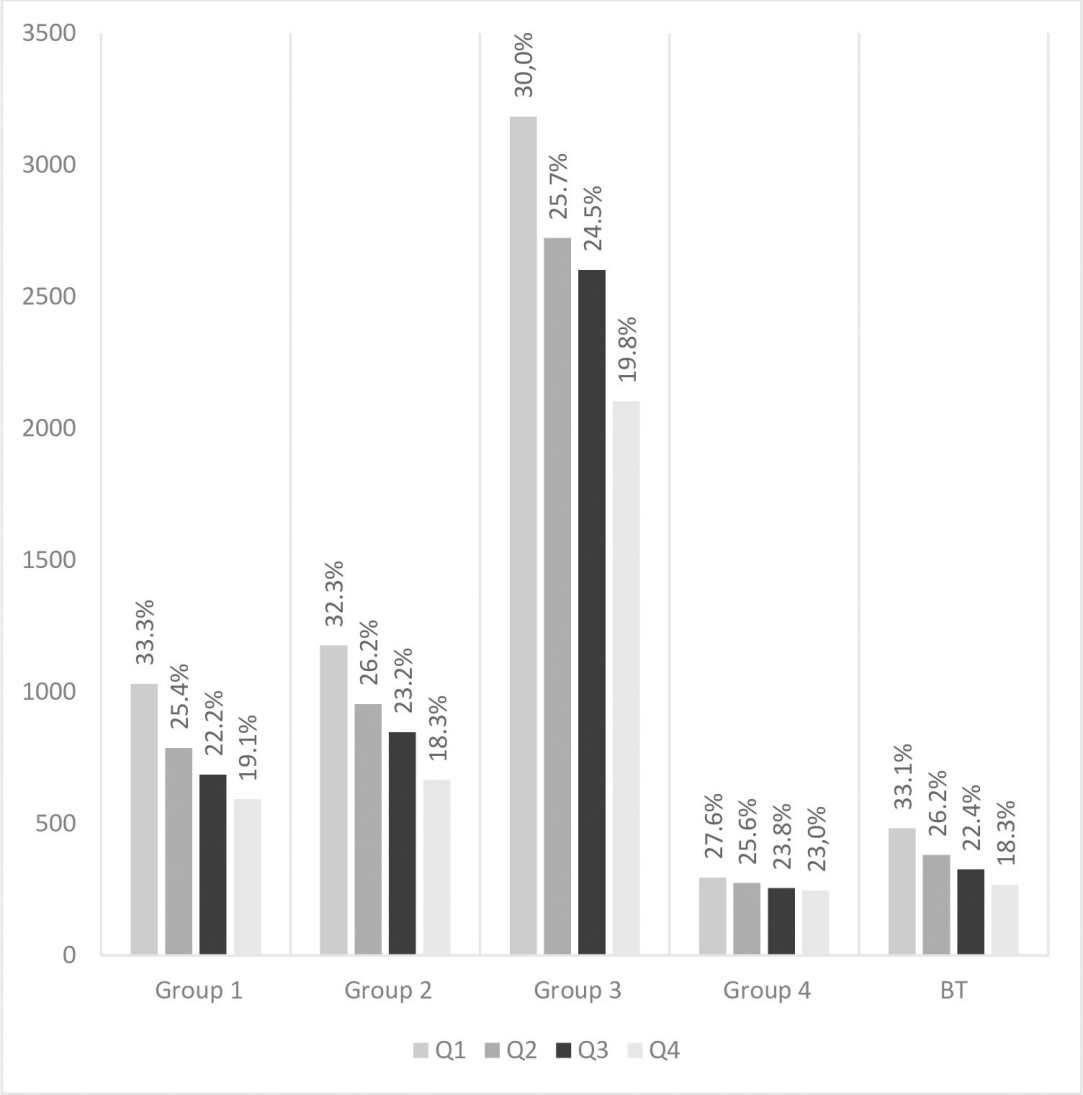
Distribution of players by quarter of birth in Groups 1–4 and BT.

When analyzing the prevalence of the RAE, a significant predominance of soccer players born in Q1 over those born in Q4 was revealed in all groups except for Group 4, which included countries with the lowest representation in the Champions League (Fig 2).

In all groups except Group 4, the proportion of players born in Q1 was 30% or more (Table 1). The highest percentage of players born in Q4 (23%) was observed in Group 4. In all other groups, this did not exceed 20% (Table 2). The highest percentage of early-born players was observed in Group 1 and in the BT group (33%) which represented the most competitive groups.

**Table 1:** Distribution of “early-born” players in various groups.

	Groups				
	1	2	3	4	BT
**Early-born (Q1)**	1031 (33.28%)	1177 (32.29%)	3184 (30.00%)	296 (27.56%)	483 (33.08%)
**Q2-Q4**	2067 (66.72%)	2468 (67.71%)	7428 (70.00%)	778 (72.44%)	977 (66.92%)
**All**	3098	3645	10612	1074	1460

**Table 2:** Distribution of “late-born” players in various groups.

	Groups				
	1	2	3	4	BT
**Late-born (Q4)**	593(19.14%)	666 (18.27%)	2103 (19.82%)	247 (23.00%)	268 (18.34%)
**Q1-Q3**	2505 (80.86%)	2979 (81.73%)	8509 (80.18%)	827 (77.00%)	1192 (81.66%)
**All**	3098	3645	10612	1074	1460

**Fig 3:**
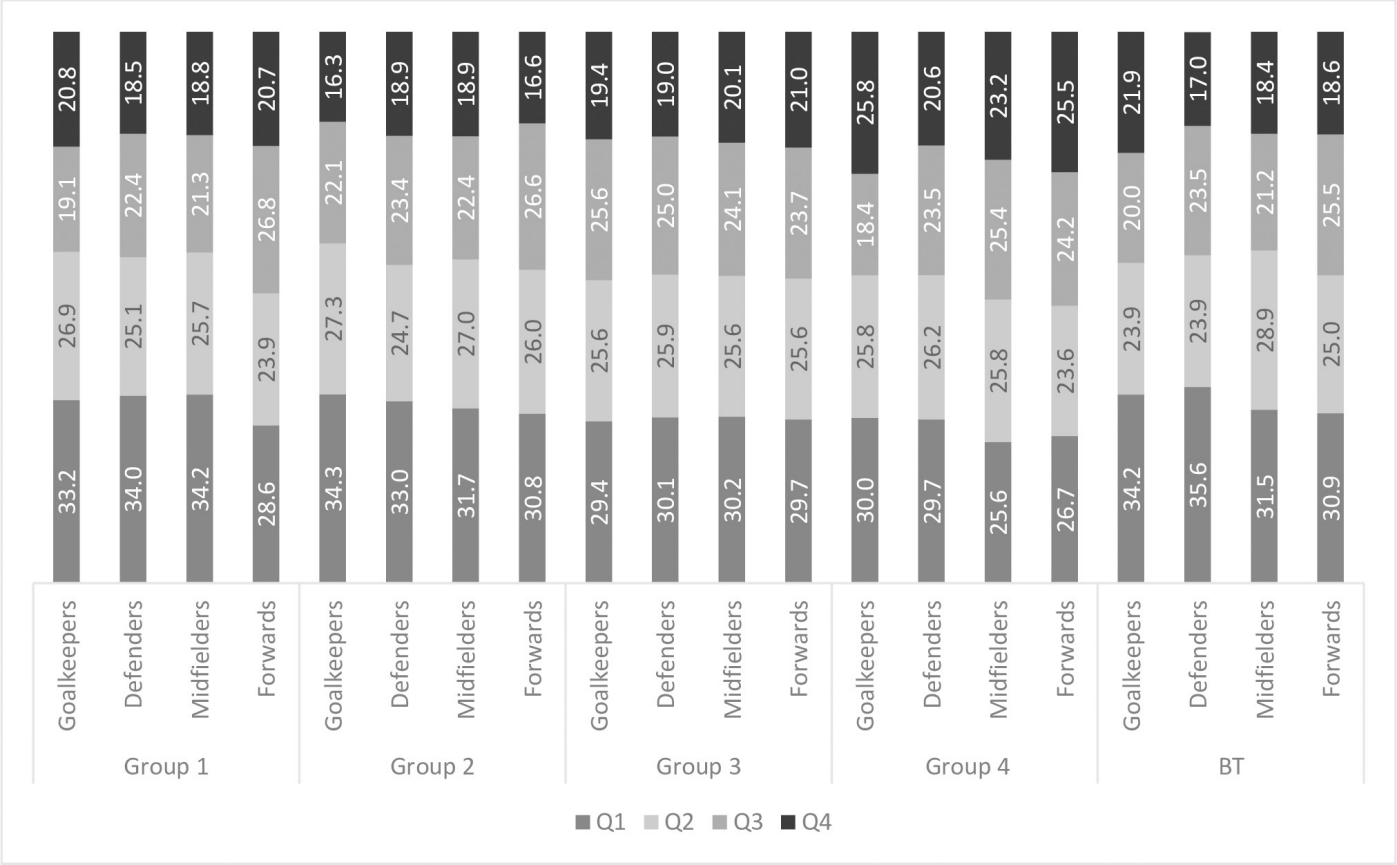
Relative Age Effect by playing position (%).

The strongest RAE was observed in defenders, where 31.3% (1844 players) were born in Q1 and 19% (1116 players) were born in Q4. The weakest RAE was observed in the forwards, where 29.5% (781 players) were born in Q1 and 20.4% (539 players)–in Q4. The RAE was observed in every analyzed group across all positions. Analysis of the dates of birth of players playing in different positions showed that for each position there was a majority of “early-born” players (Fig 3).

**Fig 4:**
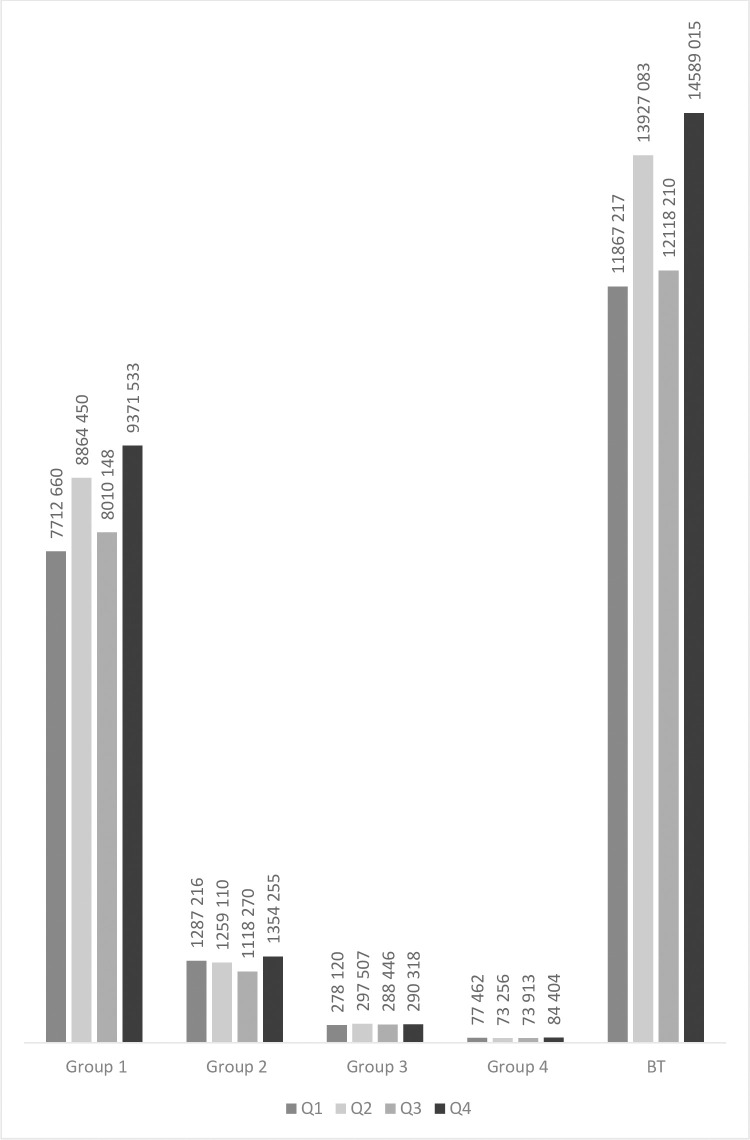
The association between a player’s quarter of birth and market value.

The mean market value was the highest in players born in Q4 (€2,200,056) and the lowest in players born in Q3 (€1,815,839). The mean market value for players born in Q1 was €1,991,380, and €2,081,436 in Q2. (Fig 4). In the most competitive groups (Groups 1, 2, and BT), the highest mean market value was observed for players born in Q4 in BT (€14,589,015).

**Fig 5:**
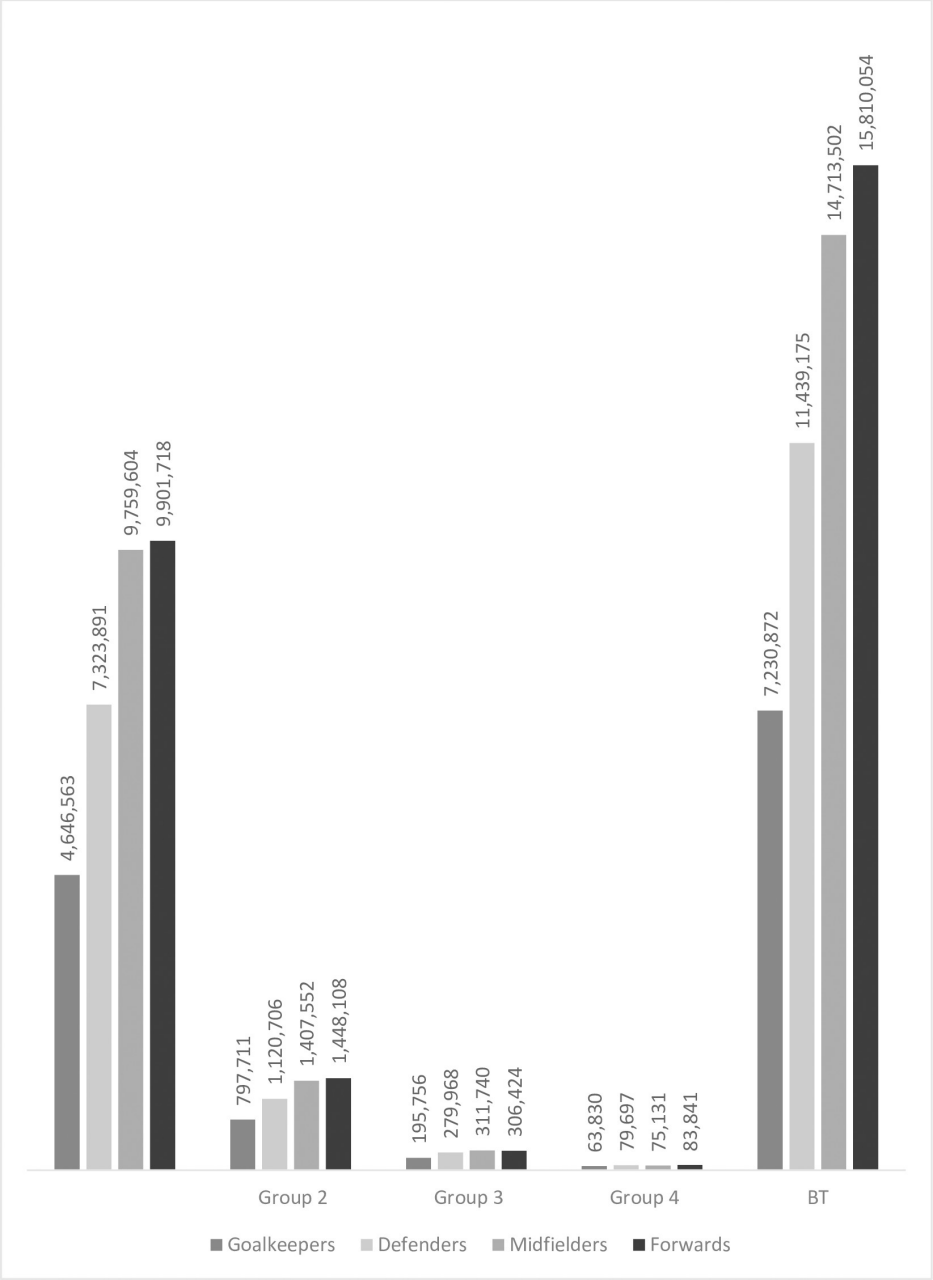
Relationship between the player’s playing position and transfer fee.

Forwards and midfielders had the highest market value in all **groups** (Fig 5).

### Characteristics of the number, player’s playing position, and the market value of soccer players of the analyzed groups

#### Group 1

Group 1 was composed of 114 teams with 3,098 players in total. Of these, 33.3% (1,031) were born in Q1, 25.4% (787) in Q2, 22.2% (687) in Q3 and 19.1% (593) in Q4. Goal-keepers made up 346 of the players, 1,040 were defenders, 1,286 were midfielders, and 426 were forwards. In Group 1, most players were defenders and midfielders (34% and 34.2%, respectively) born in Q1 (“early-born”). Players born in Q4 (“late-born”) were mainly goal-keepers and forwards (20.8% and 20.7%, respectively). The mean **market value** of players was €8,389,870.

#### Group 2

Group 2 was composed of 143 teams with 3,645 players in total. Of these, 32.3% (1,177) were born in Q1, 26.2% (954) in Q2, 23.3% (848) in Q3 and 18.3% (666) in Q4. Goalkeepers made up 417 of the players, 1,139 were defenders, 1,582 were midfielders, and 507 were forwards. In Group 2, most players were goal-keepers and defenders (34.3% and 33%, respectively) born in Q1 (“early-born”). Players born in Q4 (“late-born”) were mainly defenders and midfielders (18.9% each). The mean marker value of Group 2 players was €1,253,241.

#### Group 3

Group 3 was composed of 429 teams with 10,612 players in total. Of these, 30% (3,184) were born in Q1, 25.7% (2,723) in Q2, 24.5% (2,602) in Q3 and 19.8% (2,103) in Q4. Goal-keepers made up 1,192 of the players, 3,365 were defenders, 4,509 were midfielders, and 1,546 were forwards. Approximately 30% of the players in each playing position were born in Q1 (“early-born”). Players born in Q4 (“late-born”) were mainly forwards (21%). The mean market value of Group 3 players was €288,037. Midfielders born in Q2 and forwards born in Q3 had the highest market values of €334,830 and €330,387, respectively.

#### Group 4

Group 4 was composed of 45 teams with 1,074 players in total. Of these, 27.6% (296) were born in Q1, 25.6% (275) in Q2, 23.8% (256) in Q3 and 23% (247) in Q4. Goal-keepers made up 120 of the players, 340 were defenders, 449 were midfielders, and 165 were forwards. Players born in Q1 (“early-born”) were mainly goal-keepers and defenders (30% and 29.7%, respectively). Players born in Q4 (“late-born”) were mainly goal-keepers and forwards (25.8% and 25.5%, respectively). In this group, the RAE was the weakest. The mean market value of Group 4 players was €77,062. Defenders born in Q4 and forwards born in Q1 had the highest market values of €114,423 and €95,455, respectively.

#### Group «Best teams»

Group **BT** was composed of 50 teams with 1460 players in total. Of these, 33.1% (483) were born in Q1, 26.1% (382) in Q2, 22.4% (327) in Q3 and 18.4% (268) in Q4. Goal-keepers made up 155 of the players, 494 were defenders, 623 were midfielders, and 188 were forwards. Players born in Q1 (“early-born”) were mainly goal-keepers and defenders (34.2% and 35.6%, respectively). Players born in Q4 (“late-born”) were mainly goal-keepers (21.9%). The mean **market value** of Group **BT** players was €12,963,191.

### Comparison between groups

When comparing the dates of birth of players from different groups, there was a correlation between the level of competition and the number of “late-born” players. As the level of participation increases, the number of players born in Q4 decreases.

Table 3 shows the differences between the number of “late-born” players in different groups. In all cases where the difference was statistically significant (p <0.05), there were more “late-born” players in the less elite groups.

**Table 3:** Differences between the number of ‘late-born’ players in various groups (p values)—Chi-square.

ALL PLAYERS	Group 1	Group 2	Group 3	Group 4	Group BT
**Group 1**		0.36	0.40	**p = 0.007** **OR = 1.26** **95% CI 1.07–1.49**	0.53
**Group 2**			**p = 0.038** **OR = 1.10** **95% CI 1.00–1.22**	**p = 0.001** **OR = 1.34** **95% CI 1.13–1.58**	0.94
**Group 3**				**p = 0.013** **OR = 1.21** **95% CI 1.04–1.40**	0.18
**Group 4**					**p = 0.005** **OR = 0.75** **95% CI 0.62–0.91**
**Group BT**					

Table 4 shows the differences in the number of "early-born" players in different groups. In all cases where the difference was statistically significant (p <0.05), there were more “early-born” players in the more elite groups.

**Table 4:** Differences between the number of “early-born” players in various groups (p values)—Chi-square.

ALL PLAYERS	Group 1	Group 2	Group 3	Group 4	Group BT
**Group 1**		p = 0.39	**p = 0.001** **OR = 0.86** **95% CI 0.79–0.94**	**p = 0.001** **OR = 0.76** **95% CI 0.65–0.89**	p = 0.90
**Group 2**			**p = 0.01** **OR = 0.90** **95% CI 0.83–0.98**	**p = 0.003** **OR = 0.79** **95% CI 0.69–0.93**	p = 0.59
**Group 3**				p = 0.095	**p = 0.017** **OR = 1.15** **95% CI 1.03–1.30**
**Group 4**					**p = 0.003** **OR = 1.30** **95% CI 1.09–1.54**
**Group BT**					

Market value did not differ significantly between players born in Q1-4, including between “early-born” (Q1) and “late-born” (Q4) players (p> 0.05). Also, there was no significant difference in market value between players of different playing positions, born in different quarters. (Table 5). When analyzing the market value of all soccer players, it was found that forwards born in the Q4 were the most expensive players.

**Table 5:** Differences between market value across Q1-4—Kruskal-Wallis test.

	Statistics	Q1	Q2	Q3	Q4	p-value
**All players**						
	Mean	3 631 315.6	4 576 169.4	4 671 688.6	5 243 942.0	0.95
	SD	4 955 537.26	6 011 980.19	5 794 754.18	6 620 312.58	
	Min.	77 462	73 256	73 913	84 404	
	Max.	11 867 217	13 927 083	12 118 210	14 589 015	
**Goal-keepers**	Mean	2 547 860.6	3 364 190.8	1 893 253.8	2 522 341.8	0.95
	SD	3 048 045.77	4 305 097.70	2 075 306.93	3 258 930.76	
	Min.	70 455	68 750	90 625	42 188	
	Max.	6 622 500	10 025 714	4 820 833	7 375 000	
**Defenders**	Mean	3 755 068.2	4 542 361.0	3 672 883.2	4 458 000.2	0.89
	SD	4 713 027.24	5 896 672.66	4 516 304.24	5 600 505.28	
	Min.	61 000	82 558	77 717	114 423	
	Max.	10 504 360	13 438 478	9 904 783	12 732 229	
**Midfielders**	Mean	5 122 658.6	5 352 984.0	5 119 504.6	5 505 792.0	0.98
	SD	6 501 088.77	6 763 524.54	6 475 764.89	6 873 286.38	
	Min.	87 755	58 654	60 119	93 229	
	Max.	14 698 684	14 825 857	14 405 303	14 922 588	
**Forwards**	Mean	4 199 277.0	5 382 842.8	5 294 129.4	8 145 623.2	
	SD	4 972 403.44	6 742 400.46	6 980 970.51	11 144 686.57	0.99
	Min.	95 455	92 045	88 158	56 579	
	Max.	11 142 105	14 681 383	15 768 617	25 539 394	

Mean market value differed significantly across various groups for all players, goalkeepers, defenders, midfielders, and forwards (p = 0.001) between each group. As expected, the highest player price was observed in group **BT**, while the lowest was observed in Group 4 (Table 6).

**Table 6:** Differences between market value across groups—Kruskal-Wallis test.

	Statistics	Group 1	Group 2	Group 3	Group 4	Group BT	p-value
**All players**	Mean	7 907 944.0	1 254 712.8	288 597.8	77 258.8	13 125 381.2	0.001
	SD	2 475 311.66	99 332.91	8 002.92	5 109.21	1 339 442.75	
	Min.	4 646 563	1 118 270	278 120	73 256	11 867 217	
	Max.	9 901 718	1 354 255	297 507	84 404	14 589 015	
**Goal-keepers**	Mean	4 510 579.5	925 038.5	194 924.5	68 004.5	7 211 011.8	0.001
	SD	927 264.15	275 244.89	24 963.26	19 872.42	2 160 921.21	
	Min.	3 250 000	577 574	158 829	42 188	4 820 833	
	Max.	5 370 989	1 145 982	213 500	90 625	10 025 714	
**Defenders**	Mean	7 383 739.2	1 139 765.0	282 999.5	83 924.5	11 644 962.5	0.001
	SD	462 463.94	134 929.60	23 429.07	22 331.53	1 705 681.20	
	Min.	6 920 415	1 021 781	267 785	61 000	9 904 783	
	Max.	7 800 785	1 325 000	317 564	114 423	13 438 478	
**Midfielders**	Mean	9 870 444.0	1 404 968.5	312 714.2	74 939,2	14 713 108.0	0.001
	SD	765 074.38	80 863.72	21 541.47	18 107.16	224 756.30	
	Min.	9 049 884	1 318 496	285 761	58 654	14 405 303	
	Max.	10 753 517	1 491 209	334 830	93 229	14 922 588	
**Forwards**	Mean	10 146 349.8	1 460 760.2	304 296.5	83 059.2	16 782 874.8	0.001
	SD	2 296 107.09	307 373.34	33 929.39	17 903.44	6 162 788.85	
	Min.	7 743 388	1 053 125	257 801	56 579	11 142 105	
	Max.	13 165 116	1 709 226	330 387	95 455	25 539 394	

Post hoc pairwise comparisons were also performed using the Dunn test with Holm adjustment which showed significant differences in market value between Groups 1 and 4, 4 and **BT,** and 3 and **BT** (Table 7).

**Table 7:** Differences between market value between groups (p values)—Dunn test with Holm adjustment.

All players	Group 1	Group 2	Group 3	Group 4	Group BT
Group 1		p = 1	p = 0.39	**p = 0.037**	p = 0.34
Group 2			p = 1	p = 0.33	p = 0.28
Group 3				p = 0.68	**p = 0.033**
Group 4					**p = 0.0013**
**Goalkeepers**	**Group 1**	**Group 2**	**Group 3**	**Group 4**	**Group 5**
Group 1		p = 1	p = 0.29	**p = 0.025**	p = 0.47
Group 2			p = 1	p = 0.33	p = 0.36
Group 3				p = 0.68	**p = 0.048**
Group 4					**p = 0.0021**
**Defenders**	**Group 1**	**Group 2**	**Group 3**	**Group 4**	**Group 5**
Group 1		p = 1	p = 0.39	**p = 0.037**	p = 0.34
Group 2			p = 1	p = 0.33	p = 0.27
Group 3				p = 0.68	**p = 0.033**
Group 4					**p = 0.0013**
**Midfielders**	**Group 1**	**Group 2**	**Group 3**	**Group 4**	**Group 5**
Group 1		p = 1	p = 0.39	**p = 0.037**	p = 0.34
Group 2			p = 1	p = 0.33	p = 0.28
Group 3				p = 0.68	**p = 0.033**
Group 4					**p = 0.013**
**Forwards**	**Group 1**	**Group 2**	**Group 3**	**Group 4**	**Group BT**
Group 1		p = 1	p = 0.34	**p = 0.031**	p = 0.40
Group 2			p = 1	p = 0.33	p = 0.32
Group 3				p = 0.68	**p = 0.040**
Group 4					**p = 0.0017**

## Discussion

The study aimed to examine the prevalence of the RAE and any differences in the level of competition and playing position. This study also analyzed the relationship between player’s birth quarter and market value across 18,429 soccer players from 731 top division teams playing in 54 European Championships. Overall, the RAE was widely prevalent in the most competitive senior soccer teams and leagues in Europe regardless of playing position. The number of players born in Q1 decreased as the level of competition decreased, where the highest percentage of these players was observed in clubs that are among the top 50 ranked in UEFA listings or competed in the most prestigious European championships. The RAE was less pronounced in teams that participated in the lower competitive championships. There was no difference in market value between players of the same playing position and level of competition born in different quarters. Although, the most expensive soccer players in the European championships were late-born forwards, players from various groups differed in their market value.

The RAE has been previously described in various groups of soccer players with a different distribution pattern depending on several factors, including competitive level, age, playing position, and nationality [26,27]. According to multiple studies in soccer, the RAE is most pronounced in elite young male soccer players (under 18 years), and it can significantly influence the future career of these young players [28–30]. Furthermore, in German soccer, which is regarded as one of the most competitive in the world, “early-born” players have a greater probability of developing into professional player [31]. While in French soccer, also considered one of the leading soccer environments in the world, “late-born” players are more likely to drop out than “early-born” players [32]. These results are consistent with our findings.

The wide spread of the RAE in elite European youth and adult soccer over the past decade may be secondary to the increase in the popularity of soccer and, the differences in the level of competition, as well as the processes and timing of the initial selection. Considering that currently, the primary selection in the leading European soccer academies occurs before 10 years old, it becomes evident that a difference of even a few months of age can provide a significant advantage in terms of physical performance. **Therefore, it is not surprising that relatively older and more biologically mature soccer players will have an advantage in youth soccer [33].** Gil et al. revealed statistically significant differences in anthropometry and physical performance in older pre-pubertal soccer players with an average age of 9.75 ± 0.30 years when compared to their younger counterparts [34]. Therefore, it may be reasonable to conclude that only “late-born” athletes with a more biologically mature status are likely to have the opportunity to compete with “early-born” athletes. This finding is supported by Müller et al. who analyzed the prevalence of the RAE and the degree of biological maturity in 222 male soccer participants from the UEFA European Under-9 Championship. Müller et al. results showed that the primary selection process in young international soccer appears to be related to biological maturity status and relative age. Furthermore, “late-born” children seem more likely to be selected for highly competitive soccer organizations when a more biological mature status is evident [35].

Under the existing selection system, late-maturing children may be subjected to certain discrimination. During the initial selection, the first wave of talented athletes to drop-out occurs, generally by athletes who may be temporarily physically and psychologically less developed [36–38]. The second wave of drop-out then seems to occur during the growth spurt (12 to 14 years old), when the elimination of players from many highly competitive sports organizations can take place due to the influence of different rates of biological maturation [36–38]. Young soccer players with normal or delayed biological maturation are thus discriminated against, and the advantage is towards early-maturing athletes, regardless of date of birth. This statement is well illustrated by Malina et al. who analyzed the degree of biological maturation of elite young Portuguese Academy soccer players aged 11 years old (pre-puberty), 13 to 14 years old, and 16 to 17 years old. In these age groups, the number of soccer players with different degrees of maturation varied significantly. According to this data, at 11 to 12 years old, the proportion of early- and late-maturing boys was similar (21%), while in 13 to 14 years old players it was 38% and 7% respectively, and in 15 to 16 years old players it was 65% and 2% respectively. The results of this study show that late-maturing boys are systematically excluded from elite youth soccer, and as chronological age and sport specialization increase, preference is given to “on-time” and early-maturing boys [39]. **In contrast, it should be noted that not all young soccer players born in Q1 have a physical advantage over their younger peers, however, even in these cases coaches tended to rate players born in Q1 higher [40]. Therefore, it may be assumed that the RAE contributes to the exclusion of late-born players from highly competitive youth soccer, not only due to lower physical performance but also due to factors such as behavioral variables, coaches perceptions and the training environment [41].**

The absence of the RAE in the least competitive championships (Group 4) may be due to the low level of competition in these countries in the initial selection and a large number of “local” soccer players in adult teams. Although, the market values in the most competitive groups (Groups 1, 2, and BT) were highest for players born in Q4, as the Kruskal-Wallis test showed that market value did not differ significantly between players from different positions born in Q1-Q4, as well as between "early-born" and "late-born" players. The similar market value of “early-” and “late-born” soccer players in adulthood confirms this notion, at least, in part dictates the need to ensure measures to reduce the severity of the RAE in childhood and youth soccer. Concurrently, it must be considered that despite a large number of studies reporting the negative impact of the RAE on the development programs of soccer players in many countries, its impact in European countries with a high level of soccer development from 2001 to 2011 did not decrease and thus the RAE can still be considered prevalent in all European countries [19].

The study by Salinero et al. conducted on adult soccer players also revealed the re-distribution of “early-born” players in the most competitive championships and persisted when players were classified by playing position. In the majority of groups, there were more “early-born” goal-keepers and defenders than in any other position. This may be explained by certain anthropometric requirements for soccer players in these positions (height and large muscle mass), which can lead to drop-out or changing to other playing positions at various stages of selection during specific time periods. In our study, however, this group of championships corresponds to Group 1. Like Salinero et al. we found that the percentage of players born in Q1 (33.3%) was higher than players born in other quarters of the year, with the highest percentage in defensive and midfield positions (34.0% and 34.2%, respectively) and the RAE was also most significant in these positions [42].

The prevalence of the RAE in elite adult midfield players may be associated with both a high competitive level and the requirements of modern soccer. Midfield players are required to perform large quantities of physical activity at high speed, which **in youth soccer** depends on a well-developed aerobic capacity and the degree of biological maturation [43]. Although there were no differences reported in the intra-group market value between “early-” and “late-born” players, while the most expensive soccer players were forwards born in Q4, which is also consistent with Romann et al. in older age group players. In the study by Romann et al. based on data from Transfermarkt.com, the prevalence of the RAE was studied in 2,000 of the most expensive soccer players aged 19 to 23 years old. In all age categories, the elevated prevalence of soccer players born in Q1 was also revealed. However, this tendency was not evident when examining by playing position. An important result of this study was the correlation between the change in the market value of “early-” and “late-born” soccer players and the increase of age. In the 19 years old age group, the most expensive soccer players were born in Q1, and players aged 21 to 23 years old were born in Q4 [44].

In the study by Doyle et al. no data indicated the different values of the most successful young soccer players. The authors investigated the relationship between a player’s market value and date of birth using data on the top 1,000 professionals and UEFA U19 Youth League players. They found no difference between the market value of “early-born” players and “late-born” [19]. In support of Doyle et al. our results were unable to report any differences in market value between players born in different quarters of the year within their respective country groups and competitive level. This trend was observed across all groups examined. Interestingly, despite the lack of statistically significant results, the total value of players born in Q4 exceeded the total value of players born in other quarters of the year. Similar results were obtained by Salinero et al. who analyzed the birth quarter of players from the top five European leagues (England, Italy, Germany, France, and Spain). They found that players born in Q1 were over-represented compared to players born in the other three quarters [42]. Furthermore, in the study by Fumarco et al. they reported possible reasons for the greater success of “late-born” athletes at the adult elite level in the National Hockey League [45]. The first explanation provided was based on psychological stability, where “late-born” players were more psychologically stable and motivated, competing with older players. This is considered the ’underdog’ hypothesis, in which relatively younger players are believed to benefit from more competitive play with their older counterparts [45]. The underdog phenomenon might also be present in soccer, where Cumming et al. showed that late-maturing players appear to possess a psychological advantage in academy soccer [37]. However, given the published data on the influence of biological maturation status and the possibility of selection into highly competitive sports organizations and maintaining this selection, this theory, at least in relation to children and elite youth soccer players, should be considered with caution [35,39,46]. The second explanation regarding biological status assumes that the selected “late-born” players are more talented, which allows them to compete with the older more developed (physically and psychologically) players [45].

In this regard, the main task of coaches and scientists should be to develop and implement measures to reduce the discriminatory influence of the RAE and the biological maturation status when screening and selecting less mature and “late-born” soccer players. Furthermore, the development of physiological and psychological assessments, considering the chronological and biological age of soccer players may allow the identification of talent to be un-biased.

The limitations of our study included the data collection procedure as this was performed from an open, online database. Even though Transfermarkt.com has previously shown to provide a reliable estimate of the market value of players [23], it is still limited. For example, it does not include female soccer players, thus, estimation of RAE among female players cannot be performed based on Transfermarkt data. Among these are the vast number of evaluations of top league players compared to minor league players, and the criteria used for player evaluation. The dependence on a high number of assessments, which require several matches to provide an adjusted market value (between 6 and 12 months) is a limitation [47,48]. Furthermore, the cross-sectional design is also a limitation.

Therefore, it can be summarized that “late-born” soccer players are not less successful, but are potentially exposed to selection bias at different stages in highly competitive sports organizations, such as soccer, where the most important physical qualities are strength and speed. In this regard, it is understandable why the “late-born” soccer players can be graded lower in adolescence (when the status of biological maturation is playing a significant role) compared with “early-born” players. Future studies should aim to examine the influence of factors such as player injury history on the market value as well as measures aiming to mitigate the discriminatory effects of selection bias based on the RAE including education of relevant stakeholders in soccer academies.

## Conclusions

The RAE is currently prevalent in all of the most competitive senior soccer leagues and teams in Europe regardless of playing position. There are no significant differences in market value between players of the same playing position and competitive level when born in different quarters. The most expensive soccer players in the European championships are forwards born in Q4. These data may indicate the under-representation of “late-born” soccer players in youth, and then consequently in adult soccer, is not associated with lower talent, but with other factors, possibly based on physiological characteristics and socio-cultural factors. Further measures are needed to mitigate the discriminatory effects of selection bias based on the RAE.
